# HIV-1 infection activates endogenous retroviral promoters regulating antiviral gene expression

**DOI:** 10.1093/nar/gkaa832

**Published:** 2020-10-06

**Authors:** Smitha Srinivasachar Badarinarayan, Irina Shcherbakova, Simon Langer, Lennart Koepke, Andrea Preising, Dominik Hotter, Frank Kirchhoff, Konstantin M J Sparrer, Gunnar Schotta, Daniel Sauter

**Affiliations:** Institute of Molecular Virology, Ulm University Medical Center, Ulm 89081, Germany; Molecular Biology Division, Biomedical Center, Ludwig-Maximilians-University Munich, Planegg-Martinsried 82152, Germany; Institute of Molecular Virology, Ulm University Medical Center, Ulm 89081, Germany; Sanford Burnham Prebys Medical Discovery Institute, La Jolla, CA 92037, USA; Institute of Molecular Virology, Ulm University Medical Center, Ulm 89081, Germany; Institute of Molecular Virology, Ulm University Medical Center, Ulm 89081, Germany; Institute of Molecular Virology, Ulm University Medical Center, Ulm 89081, Germany; Institute of Molecular Virology, Ulm University Medical Center, Ulm 89081, Germany; Institute of Molecular Virology, Ulm University Medical Center, Ulm 89081, Germany; Molecular Biology Division, Biomedical Center, Ludwig-Maximilians-University Munich, Planegg-Martinsried 82152, Germany; Institute of Molecular Virology, Ulm University Medical Center, Ulm 89081, Germany

## Abstract

Although endogenous retroviruses (ERVs) are known to harbor *cis*-regulatory elements, their role in modulating cellular immune responses remains poorly understood. Using an RNA-seq approach, we show that several members of the ERV9 lineage, particularly LTR12C elements, are activated upon HIV-1 infection of primary CD4^+^ T cells. Intriguingly, HIV-1-induced ERVs harboring transcription start sites are primarily found in the vicinity of immunity genes. For example, HIV-1 infection activates LTR12C elements upstream of the interferon-inducible genes *GBP2* and *GBP5* that encode for broad-spectrum antiviral factors. Reporter assays demonstrated that these LTR12C elements drive gene expression in primary CD4^+^ T cells. In line with this, HIV-1 infection triggered the expression of a unique *GBP2* transcript variant by activating a cryptic transcription start site within LTR12C. Furthermore, stimulation with HIV-1-induced cytokines increased GBP2 and GBP5 expression in human cells, but not in macaque cells that naturally lack the *GBP5* gene and the LTR12C element upstream of *GBP2*. Finally, our findings suggest that GBP2 and GBP5 have already been active against ancient viral pathogens as they suppress the maturation of the extinct retrovirus HERV-K (HML-2). In summary, our findings uncover how human cells can exploit remnants of once-infectious retroviruses to regulate antiviral gene expression.

## INTRODUCTION

Endogenous retroviruses (ERVs) are the result of infectious viruses that have become an integral part of the human genome and are inherited in a Mendelian fashion (1). They come in a variety of shapes and sizes, ranging from full-length proviral genomes to short provirus fragments. Full-length ERVs comprise the typical retroviral genes *gag*, *pol* and *env* that are flanked by long terminal repeat (LTR) promoters. Usually, ERV gene expression is suppressed by epigenetic modifications mediated by KRAB zinc finger proteins and other host repressors (2). Furthermore, ERVs accumulated numerous inactivating mutations during primate evolution that preclude the production of replication-competent viral particles in humans. In many cases, recombination of homologous LTR sequences even resulted in a complete loss of retroviral genes, leaving only the LTR promoter behind (3). These so-called solo-LTRs account for approximately 85% of all endogenous retroviral repeats in the human genome (4).

The fixation of hundreds of thousands of solo-LTRs and other ERV sequences in the human genome may be explained by their success as viral parasites that outpace the ability of the host species to purge them from the genome. Although most ERVs in the human genome may have neutral effects on host fitness because they are transcriptionally silent, they can serve as an important source of regulatory elements and genes that may be exploited by the host organism. The paramount example of retroviral genes that have been coopted by the human host are *env*-derived syncytins, mediating cell-cell fusion during placenta development (5). In the 1950s, Barbara McClintock already proposed that transposable elements such as ERVs may also regulate cellular gene expression (6). Indeed, numerous ERV-derived LTRs harbor promoter, enhancer, silencer or insulator elements modulating the transcription of cellular genes (7). In line with this, transposable elements have been estimated to contribute ∼20% of all transcription factor binding sites in humans (8) and frequently harbor conserved transcription start sites (9). Recent evidence suggests that HERV LTRs are particularly important in regulating pathways involved in immunity, inflammation and apoptosis (10–13). For example, LTR elements of the endogenized gamma-retrovirus MER41 harbor STAT1 binding sites and enhance the expression of AIM2, IFI6, APOL1 and other immunity factors in response to IFN-γ (10).

Notably, infections with exogenous viruses such as the Human immunodeficiency virus type 1 (HIV-1), Influenza A virus (IAV), Dengue virus (DENV), Hepatitis B Virus (HBV) or Epstein Barr virus (EBV) are known to trigger the activation of ERVs (14–18). For example, HIV-1 infection results in the transcription and translation of HERV-K (HML-2) loci, representing the youngest ERV family in humans (14,19–25). Several follow-up studies focused on the induction of cellular and humoral immune responses against HERV-K (HML-2) proteins in HIV-1 infected individuals (26–30) and a possible interference of ERV proteins with HIV (31–33). In contrast, the impact of HIV-1 infection on regulatory ERV elements and their downstream effects on cellular gene expression remained unclear.

Using an unbiased approach (RNA-seq), we here show that *ex vivo* HIV-1 infection of primary CD4^+^ T cells induces the activation of multiple solo-LTRs belonging to the ERV9 lineage of endogenous retroviruses. This includes so-called LTR12C repeats in the vicinity of genes involved in antiviral immunity. The enrichment of LTR12C elements was particularly pronounced among HIV-1-induced ERVs harboring transcription start sites. By combining RNA-seq with promoter reporter assays, we identify two HIV-1 responsive LTR12C repeats that act as promoters for guanylate-binding proteins 2 and 5 (GBP2 and 5). These two IFN-inducible GTPases exert broad antiviral activity, inhibiting the replication of HIV-1, measles virus, Zika virus and other viral pathogens (34,35). A comparative analysis of different primate species revealed that the LTR12C repeats upstream of *GBP2* and *GBP5* can only be found in greater apes and are associated with increased cytokine responsiveness of these antiviral genes. Furthermore, we demonstrate that GBP2 and GBP5 reduce the infectivity of HERV-K (HML-2) pseudoparticles, suggesting that these two LTR-induced host factors have already been active against ancient viral pathogens. In summary, our findings illustrate how human cells make use of retroviral relics to improve innate immune responses against current viral pathogens.

## MATERIALS AND METHODS

### Cell culture

Human embryonic kidney 293T (HEK293T) cells (authenticated and obtained from the American Type Culture Collection [ATCC]) were first described by DuBridge *et al.* (36) and cultured in Dulbecco's modified Eagle medium (DMEM) containing 10% heat-inactivated fetal calf serum (FCS) plus 2 mM glutamine, 100 μg/ml streptomycin and 100 units/ml penicillin. HEK293T cells were tested for mycoplasma contamination every three months. Only mycoplasma negative cells were used for this study. Crandell-Rees Feline Kidney (CRFK) cells were cultured in Dulbecco's modified Eagle's medium (DMEM) containing 10% heat-inactivated fetal calf serum (FCS) plus 2 mM glutamine, 100 μg/ml streptomycin and 100 units/ml penicillin.

Human and rhesus macaque PBMCs were isolated using lymphocyte separation solution (Biocoll separating solution; Biochrom). PBMCs from healthy human donors were obtained from lymphocyte concentrates from 500 ml whole blood (blood bank Ulm) while macaque PBMCs were isolated from blood purchased from Silabe (Strasbourg). CD4^+^ T cells were negatively isolated using the RosetteSep Human CD4^+^ T Cell Enrichment Cocktail (Stem Cell Technologies) according to the manufacturer's instructions. Primary cells were cultured in RPMI-1640 medium containing 10% fetal calf serum (FCS), 2 mM glutamine, 100 μg/ml streptomycin, 100 units/ml penicillin, and 10 ng/ml interleukin 2 (IL-2) at 37°C in a 5% CO_2_ atmosphere. Before infection, cells were stimulated for 3 days with 1 μg/ml phytohemagglutinin (PHA).

### Proviral constructs

Infectious molecular clones of HIV-1 CH293, CH077 and STCO1 have been described previously (37,38).

### Generation of virus stocks

HEK293T cells were co-transfected in a six-well format with proviral constructs (5 μg) and an expression plasmid for the vesicular stomatitis virus glycoprotein (pHIT_VSV-G; 1 μg) (39) using the calcium phosphate method. Two days post-transfection, cell culture supernatants were harvested and cleared by centrifugation (1700 g, 4 min, 4°C, Centrifuge 5417C (Eppendorf, Hamburg, Germany), fixed-angle rotor F-45-30-11). In some cases, virus stocks were concentrated 20-fold via ultracentrifugation (96 325 g, 120 min, 4°C, UC OptimaTM L-80 XP Ultracentrifuge (Beckman Coulter, Brea, CA, USA), Swinging-Bucket Rotor SW Ti-32) before infection of primary cells.

### Transcriptome sequencing

RNA was isolated and sequenced as part of a previous study (40) (GEO accession GSE117655). Briefly, RNA sequencing libraries were generated from 150 ng of RNA using Illumina's TruSeq Stranded mRNA Sample Prep Kit following the manufacturer's instructions, modifying the shear time to 5 min. RNA libraries were multiplexed and sequenced with 50 base pair (bp) single reads (SR50) to a depth of ∼30 million reads per sample on an Illumina HiSeq4000.

### Read mapping

All annotations and pipelines were based on human genome version hg38. Raw reads were mapped to the human genome using TopHat (41). TopHat aligns RNA-seq reads using short read aligner Bowtie (42). Using TopHat, we filtered out all low quality reads with a low mapping score (MAPQ < 20). Using Homer (43), multi-mapped and uniquely mapped reads were divided into separate pools. Later, multi-mapped reads were used to create an expression matrix for ERV families, and uniquely mapped reads were used to create expression matrices for single ERV loci and genes.

### Generation of expression matrices

To create an expression matrix for ERV loci, we used the software Homer (43). As a reference file for individual ERV genomic positions, we used a RepeatMasker-based annotation table of repetitive elements (44). Specifically, all loci except ERV elements were removed from a RepeatMasker table, and each ERV element was given a unique number. To create a reference for transcription start site-containing ERV elements, we used FANTOM bed files with genomic positions of CAGE signals combined from several hundred human samples (45). Using bedtools, we overlapped genomic positions of individual ERV loci from RepeatMasker and CAGE signals from the FANTOM project. All ERV loci containing CAGE signals within their sequence were kept as CAGE-containing individual ERV loci. As a reference for repeat families, we used the in-build HOMER repeat family annotation, which was loaded from UCSC. Using Homer, we normalized the total number of mapped reads to 10 million reads per experiment. Then, the normalized expression matrix was loaded into R software.

### Differential expression analysis

The log2-fold normalized read counts were calculated for each ERV locus. Then, the mean of all four donors was calculated for each ERV locus. For each HIV-1 clone, log2-fold changes between the mean expression in HIV-1 infected cells and mock infected cells were calculated. To obtain *P* values for individual ERV loci and families, a *t*-test was applied to four replicates (i.e. donors) in uninfected controls and four replicates in HIV-1 infected samples for each locus/family. To calculate adjusted *P* values, a false discovery rate (FDR) test was applied (46). To determine differential gene expression, gene expression matrixes were calculated and normalized by Homer (to 10 million mapped reads per experiment) and loaded into R software.

### GREAT analysis

Pathways that may be *cis*-regulated by HIV-1-induced ERVs were determined using GREAT version 4.0.4 (species assembly: hg38; association rule: basal + extension: 5000 bp upstream, 1000 bp downstream, 1 000 000 bp max extension, curated regulatory domains included) (http://great.stanford.edu/public/html/) (47).

### Reporter plasmids

To generate promoter reporter vectors, LTR12C loci and their upstream regions were synthesized (BaseClear B.V., Netherlands) and fused with the firefly luciferase or blue fluorescent protein (*BFP*) gene using overlap extension polymerase chain reaction (OE-PCR) and cloned into pGL4.32 (Promega) via NheI/EcoRI. The promoter reporter vector harboring the 5′ LTR of HIV-1 NL4–3 (corresponding to nucleotides 1–789 of the HIV-1 reference genome MA255543.1) was generated accordingly. Enhancer reporter vectors were generated by inserting the respective LTR12C sequences into the pGLuc Mini-TK 2 *Gaussia* enhancer reporter plasmid (NEB) via XhoI/HindIII, upstream of the minimal promoter. All primers used for the generation of reporter constructs are listed in Supplementary Table S1 and were purchased from biomers.net. The GATA-2 responsive *iASPP* promoter reporter plasmid has been previously described (48).

### Promoter reporter assays

Promoter activity was determined in transfected HEK293T cells or electroporated primary CD4^+^ T cells using luciferase and BFP reporter vectors, respectively. For the HEK293T cell experiments, 96-well plates were coated with poly-l-lysine, and cells were seeded (20 000 cells/well) one day prior to transfection. A standard calcium phosphate transfection protocol was used for transfection. Promoter reporter constructs expressing firefly luciferase under the control of the 21221_LTR12C(*GBP2*) or 21276_LTR12C(*GBP5*) promoter were used to investigate potential promoter activity of LTR12C. A reporter construct without a promoter insert served as negative control. pTAL *Gaussia* luciferase was used for normalization. Enhancer reporter constructs expressing *Gaussia* luciferase under the control of a minimal herpes simplex virus (HSV) thymidine kinase promoter, either alone or in combination with 21221_LTR12C or 21276_LTR12C were used to investigate the potential enhancer activity of LTR12C repeats. pTAL firefly luciferase was used for normalization. Reporter vectors and control vectors were used in a ratio of 20:1. To investigate the effect of HIV-1 or GATA-2 on LTR12C activity, HEK293T cells were co-transfected with LTR12C firefly luciferase reporter vectors, the *Gaussia* luciferase control plasmid and either an expression plasmid for GATA-2 (48) or a proviral construct of HIV-1 STCO-1. A GATA-2 responsive *iASPP* promoter reporter plasmid (48) and an HIV-1 LTR reporter construct served as positive controls. In all cases, luciferase activity was determined two days post transfection. In order to investigate if IFN-γ responsive elements (IRE) upstream of LTR12C enhance LTR12C-driven gene expression, HEK293T cells were transfected with BFP reporter constructs harboring either LTR12C alone or in combination with the respective upstream sequence. Cells were stimulated with the indicated amounts of IFN-γ or left untreated, and BFP MFI was quantified two days later via flow cytometry.

To monitor promoter activity in primary cells, CD4^+^ T cells were electroporated with BFP reporter constructs using the Human T Cell Nucleofector Kit (Lonza). Briefly, 6 million unstimulated or PHA stimulated CD4^+^ T cells were resuspended in 100 μl of the Human T Cell Nucleofector Solution and 1.5 μg of the plasmid, the cell/DNA suspension was transferred to a cuvette and electroporation was performed using Nucleofector I (Amaxa Biosystems). The V-24 and U-15 programmes were used for unstimulated and stimulated CD4^+^ T cells, respectively, according to the manufacturer's protocol. Reporter constructs with no promoter served as negative control. The electroporated cells were gently transferred into 500 μl RPMI culture medium without any antibiotics and transferred to 12-well plates 30 min later. Electroporated cells were stimulated with IFN-γ or IL-27 or left untreated. Two days post electroporation, cells were analyzed for BFP via flow cytometry.

### Cytokine stimulation

CD4^+^ T and HEK293T cells were stimulated with different doses of IFN-α2 (Pbl assay science), IFN-α14 (kindly provided by Kathrin Sutter, University Duisburg-Essen), IFN-γ (Sigma-Aldrich), IL-27 (R&D Systems,) or PHA (ThermoFischer Scientific) and IL-2 (Miltenyi Biotec GmbH). At different time points post stimulation, GBP expression was analyzed by qRT-PCR, Western blotting or flow cytometry.

### Western blotting

To determine protein levels in cells, HEK293T or CD4^+^ T-cells were washed in PBS, lysed in Western blot lysis buffer (150 mM NaCl, 50 mM HEPES, 5 mM EDTA, 0.1% NP40, 500 mM Na_3_VO_4_, 500 mM NaF, pH 7.5) and cleared by centrifugation at 20 800 g for 20 min at 4°C.

Lysates were mixed with protein sample loading buffer supplemented with 10% β-mercaptoethanol (for cells) or 2.5% β-mercaptoethanol (for supernatants) and heated at 95°C for 5 min. Proteins were separated on NuPAGE 4–12% Bis–Tris Gels, blotted onto Immobilon-FL PVDF membranes and stained using primary antibodies directed against GBP2 (Santa Cruz, #sc-271568; Origene, #TA500657), GBP5 (Santa Cruz, #sc-160353), HA-Tag (abcam, ab 18181), V5-Tag (Cell Signaling Technology, #13202), GAPDH (BioLegend, #607902) and Infrared Dye labeled secondary antibodies (LI-COR IRDye, #926-68070, #926-68076, #926-68074, #926-32210, #925-32219, #926-32214). Proteins were detected using a LI-COR Odyssey scanner.

### Flow cytometry

BFP reporter gene expression was monitored to determine HIV-1 LTR and LTR12C activity in transfected HEK293T cells or electroporated CD4^+^ T cells. Two days post transfection or electroporation, cells were washed in PBS with 2% FCS, fixed in 4% PFA and fluorescence was determined using a BD FACS Canto II flow cytometer. To quantify GBP protein levels, cells were permeabilized using the FIX & PERM kit (Nordic-MUbio, #GAS-002–1) according to the manufacturer's instructions. Subsequently, cells were stained using anti-GBP1 (Santa Cruz, #sc-53857), anti-GBP2 (Origene, #TA500657), anti-GBP5 (Santa Cruz, #sc-160353) and the following secondary antibodies: goat anti-rat, APC-conjugated (life technologies, #A10540), goat anti-mouse, PE-conjugated (life technologies, #P852), donkey anti-goat, Alexa Fluor 647-conjugated (life technologies, #A21447)

### qRT-PCR

Total RNA was isolated and purified from CD4^+^ T cells using the RNeasy Plus Mini Kit (QIAGEN) according to the manufacturer's instructions. Cells were homogenized by vortexing for 30 seconds. Subsequent gDNA digestion was performed using the DNA-*free* DNA Removal Kit (ThermoFisher Scientific) if necessary. The maximal amount of RNA was reversely transcribed with the PrimeScript RT Reagent Kit (Perfect Real Time) (TAKARA) using oligo dT primers and random hexamers. cDNA was subjected to quantitative real time PCR in duplex format using primer/probe sets for *GBP2*, *GBP5, IFI44L*, *OAS1* and *GAPDH* (ThermoFisher Scientific). Samples were analyzed in technical triplicates. Ct data was processed relative to the GAPDH control.

### Generation of HERV-K (HML-2) pseudovirions and determination of pseudovirion infectivity

To generate infectious HERV-K pseudovirions, HEK293T cells were co-transfected in a 6-well format with 2.5 μg pSIvec1ΔenvLuci, 1 μg pCDNA3.1_HIV-1 ZM247 rev1+2_Directional Topo, 0.5 μg pcDNA3_oricoEnvΔ659–699-V5 or pHIT VSV-G and either 1 μg pCG expression plasmid for GBP or the respective vector control. The simian immunodeficiency virus (SIVmac)-based retroviral vector pSIvec1ΔenvLuci (49), the plasmid expressing a C-terminally truncated variant of HERV-K Env (pcDNA3_oricoEnv delta659-699-V5) (49), GBP expression plasmids (35) and the HIV-1 Rev expression plasmid (pCDNA3.1_HIV-1 ZM247 rev1+2_Directional Topo) (50) have been described before. We used a C-terminally truncated variant of HERV-K Env since removal of 41 amino acids at the C-terminus has previously been shown to increase virion incorporation of Env and virion infectivity (49). Two days post transfection, cell culture supernatants were harvested, centrifuged at 6000 rpm for 10 min, and used to infect CRFK target cells. Briefly, CRFK target cells were sown in 96-well plates (9000 cells/well). The next day, cells were infected with either 100 μl or 50 μl of the virus supernatants. Two days later, infection was determined by measuring firefly luciferase activity in cell lysates using the Luciferase Assay System (Promega) according to the manufacturer's instructions. To calculate particle infectivity, total infectious virus yield was normalized to the amount of SIV p27 capsid in the culture supernatant. SIV p27 concentrations were determined by ELISA (Advance Bioscience laboratories, Inc; USA) according to the manufacturer's instructions.

### Statistical analyses

Statistical calculations were performed with a two-tailed unpaired Student's *t* test or a one sample *t* test using GraphPad Prism 7. To compare primary cells from different donors, a two-tailed paired Student's *t* test was used. *P* values ≤0.05 were considered significant. In the GREAT analyses, pathways significant by binomial test are shown.

### Sequence alignments

LTR12C nucleotide sequences were aligned using MultAlin (51).

### Ethical statement

Experiments involving human peripheral blood mononuclear cells were reviewed and approved by the Institutional Review Board (i.e. the Ethics Committee of Ulm University), and individuals and/or their legal guardians provided written informed consent prior to donating blood. All blood samples were anonymized before use. Rhesus macaque samples were purchased from Simian Laboratory Europe (SILABE) and collected from healthy captive-bred monkeys under veterinary supervision. The AAALAC-accredited site houses rhesus macaques in infrastructures that meet European standards and offers an optimal environment in terms of animal welfare.

## RESULTS

### HIV-1 infection triggers the transcription of ERV9 repeats in primary CD4^+^ T cells

**Figure 1.**
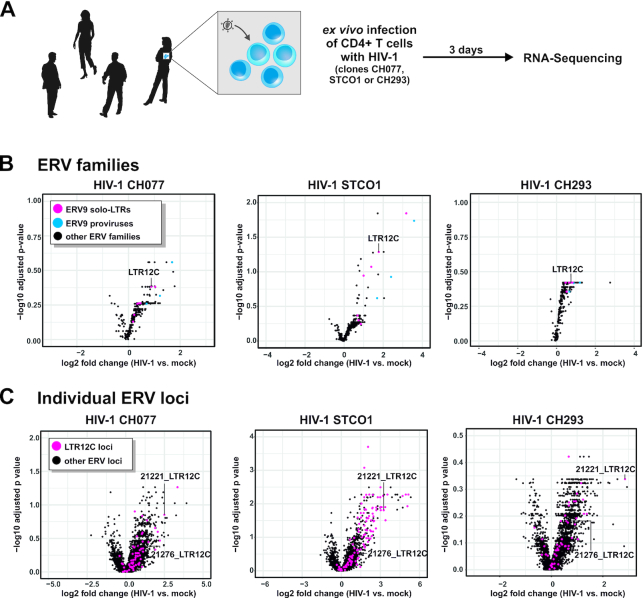
HIV-1 infection triggers ERV transcription in primary CD4^+^ T cells. (**A**) Experimental setup. CD4^+^ T cells of four healthy human donors were infected with three different clones of HIV-1 (40). Three days post-infection, cells were harvested and RNA sequencing was performed. (B, C) Differential expression of ERVs upon infection with HIV-1 CH077 (left), STCO1 (middle) or CH293 (right). (**B**) Multi-mapped reads were used to determine differential expression of ERV families. Solo-LTRs or (near) full-length members belonging to the ERV9 lineage are highlighted in pink and blue, respectively. (**C**) Uniquely mapped reads were used to determine differential expression of individual ERV loci. LTR12C loci are highlighted in pink.

To investigate the effect of HIV-1 on the transcription of endogenous retroviral elements, we took advantage of RNA sequencing data from HIV-1 infected T cells that we had obtained in the context of a previous study (40). Briefly, CD4^+^ T cells were negatively isolated from four human healthy donors (two women, two men) and *ex vivo* infected with HIV-1 (Figure 1A). We used primary CD4^+^ T cells rather than a T cell line since they represent the main target cells of HIV-1 and since ERVs are known to be aberrantly activated in immortalized cell lines (52). While previous studies used laboratory-adapted HIV-1 strains such as NL4-3 or LAI (14,20,24,25) that are known to have lost immunomodulatory activities during passage in cell culture, we selected three clones (CH293, CH077 and STCO1) that represent primary viral isolates from different stages of infection (37,38). These clones belong to the most prevalent HIV-1 M subtypes B (CH077, STCO1) and C (CH293) and represent CCR5-tropic (CH293) and dual-tropic (CH077, STCO1) viruses, respectively (37,38). Next generation sequencing of the total RNA of the HIV-1 infected CD4^+^ T cells yielded 24.6–51.9 million reads per sample (40).

**Figure 2.**
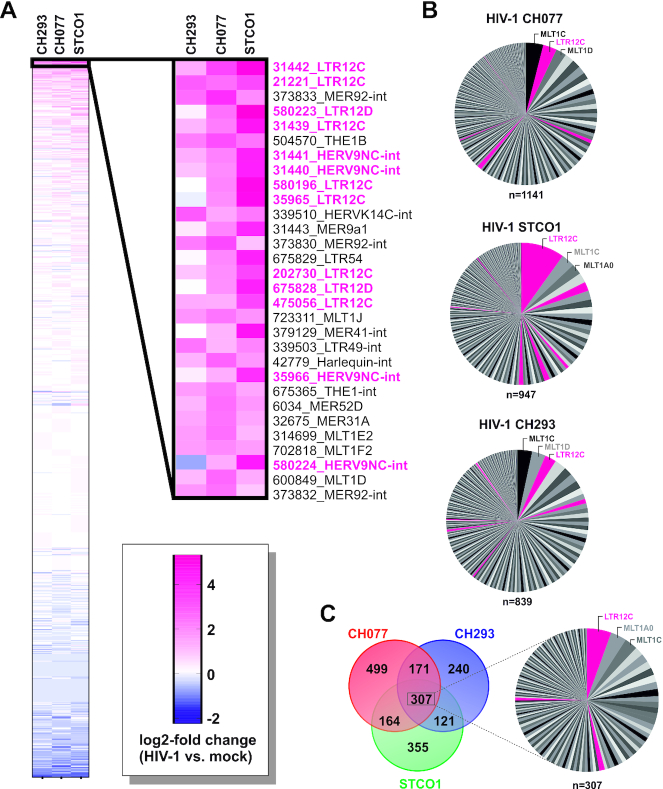
HIV-1 infection triggers the transcription of ERV9 elements in primary CD4^+^ T cells. (**A**) Heatmap illustrating log2-fold changes of individual ERV loci upon infection with HIV-1 CH293, CH077 or STCO1. The 30 most strongly upregulated ERV repeats are shown on the right. (**B**) Pie charts illustrating the number of repeats in each ERV family that were induced upon infection with HIV-1 CH077 (left), STCO1 (middle) or CH293 (right). Families belonging to the ERV9 lineage are highlighted in pink. (**C**) Venn diagram (left) illustrating the overlap of ERVs induced by HIV-1 CH077, CH293 and/or STCO1 and pie chart (right) highlighting the number of repeats in each ERV family that were upregulated by all three HIV-1 clones tested.

Using a RepeatMasker-based annotation of repetitive elements (44), we found several HERVs to be induced in HIV-1 vs. mock infected cells (Figure 1B, C). The analysis of multi-mapped reads revealed that particularly families of the ERV9 lineage were upregulated in HIV-1 infected cells compared to mock infected cells (Figure 1B). This was in line with the analysis of individual ERV loci using single-mapped reads (Figure 1C). Again, many of the most strongly upregulated ERV loci belong to the ERV9 lineage of endogenous retroviruses that was active until about 6 million years ago (53). This includes (near) full-length proviruses (e.g. HERV9NC-int, HERV9-int), as well as solo-LTRs (e.g. LTR12C, LTR12D), some of which were upregulated more than 20-fold upon HIV-1 infection (Figures 1C and 2A). Applying a log2-fold change of 0.5 as threshold (i.e. ≥41.4% increase), HIV-1 CH077, STCO1 and CH293 induced the expression of 1141, 947 and 839 endogenous retroviral repeats, respectively (Figure 2B). 307 ERVs, including 17 LTR12C repeats, were commonly upregulated by all three viruses tested (Figure 2C). Differences in the number of upregulated HERVs may reflect different infection rates as the dual-tropic viruses CH077 and STCO1 infected on average more cells (70% and 45%, respectively) than R5-tropic CH293 (30%) (40).

**Figure 3.**
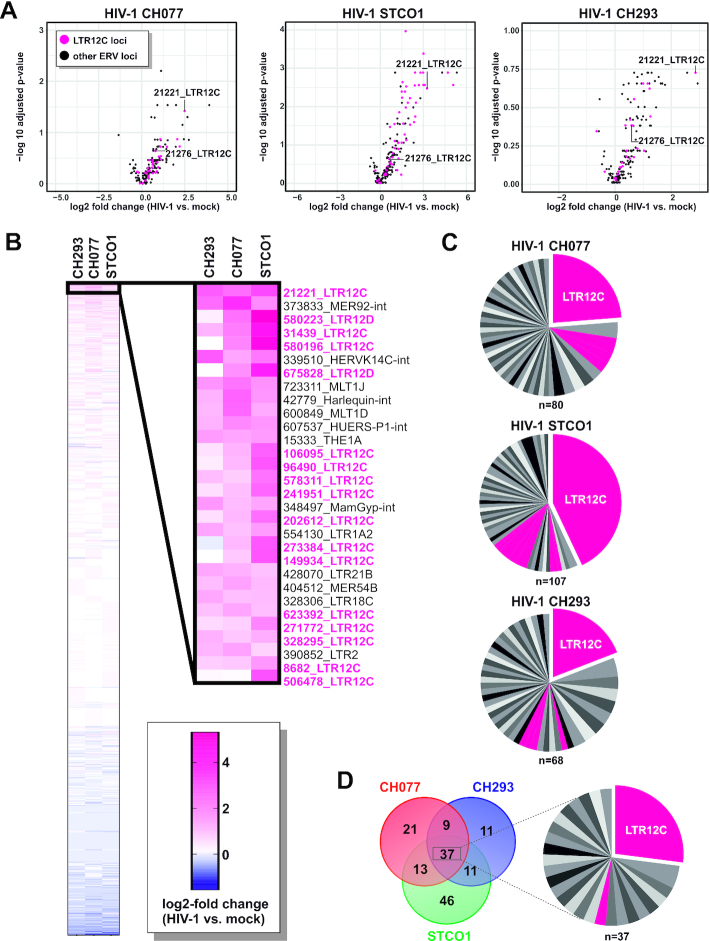
HIV-1 infection triggers the transcription of ERV9 elements harboring TSS. (**A**) Results shown in Figure 1C were CAGE-filtered to illustrate differential expression of individual ERV loci harboring transcription start sites TSS. In the CH293 volcano plot of panel (A), one outlier (463751_LTR16D) was omitted for better visualization. LTR12C loci are highlighted in pink. (**B**) Heatmap illustrating log2-fold changes of CAGE-filtered ERV loci upon infection with HIV-1 CH293, CH077 or STCO1. The 30 most strongly upregulated ERV repeats harboring previously described TSS are shown on the right. (**C**) Pie charts illustrating the number of CAGE-filtered repeats in each ERV family that were induced upon infection with HIV-1 CH077 (left), STCO1 (middle) or CH293 (right). Families belonging to the ERV9 lineage are highlighted in pink. (**D**) Venn diagram (left) illustrating the overlap of CAGE-filtered ERVs induced by HIV-1 CH077, CH293 and/or STCO1 and pie chart (right) highlighting the number of CAGE-filtered repeats in each ERV family that were upregulated by all three HIV-1 clones tested.

Importantly, the pronounced activation of ERV9 repeats was not simply a stochastic event since HIV-1-mediated induction of specific HERV family members does not correlate with the total number of repeats in each family (Supplementary Figure S1A). LTR12 repeats are well-known for their high number of transcription factor binding sites (TFBS) (54) and the presence of conserved transcription start sites (TSS) (55), suggesting that some of them may regulate cellular gene expression. We therefore used FANTOM5 CAGE profiles of several hundred human samples (45) to filter HERV elements for those harboring TSS. Upon CAGE-filtering, the enrichment for LTR12C elements among HIV-1-induced HERVs was even more pronounced (Figure 3A–C, Supplementary Figure S1B–D) and several of them were induced by all three viruses tested (Figure 3D, Supplementary Figure S1E). LTR12C repeats represented about 20–40% of all HERVs that harbor TSS and are upregulated upon HIV-1 infection (Figure 3C), although they make up <0.5% of all endogenous retroviral repeats in the human genome (56). Taken together, these findings demonstrate that HIV-1 infection of primary CD4^+^ T cells triggers the activation of ERV9 solo-LTRs harboring transcription start sites.

### HIV-1 infection activates endogenous LTR promoters in the vicinity of antiviral genes

**Figure 4.**
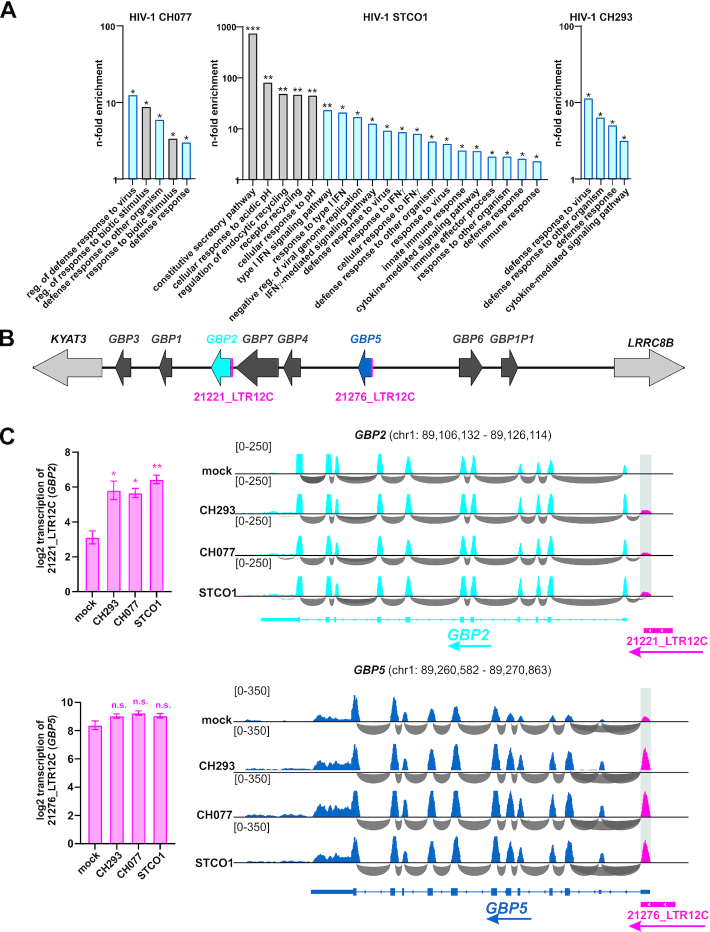
HIV-1-induced ERVs are found in the vicinity of cellular immunity genes and may regulate their expression. (**A**) Identification of cellular pathways that may be *cis*-regulated by HIV-1 induced ERVs. CAGE-filtered ERV repeats that were upregulated at least log2 0.5-fold upon infection with HIV-1 CH077, STCO1 or CH293 were analyzed using GREAT (47). Pathways significant by binomial test are shown (Binom FDR *q*-value: *q< 0.05, **q < 0.01, ***q < 0.001). Gene sets involved in immunity and infection are highlighted in blue. (**B**) Guanylate-binding protein (*GBP*) gene locus illustrating all human *GBP* genes (*GBP1–7*), a pseudogene (*GBP1P1*) and two flanking genes (*KYAT3*, *LRRC8B*). *GBP2* and *GBP5*, encoding for broad-spectrum antiviral factors (35) are shown in blue, the upstream LTR12C repeats are highlighted in pink. (**C**) Transcription of *GBP2*, *GBP5* and the respective LTR12C repeats in uninfected and HIV-1 infected CD4^+^ T cells. Representative RNA-Seq data illustrating read coverage (blue) and exon linkage (grey) are shown on the right. The mean transcription of LTR12C in all four donors for all three viruses tested (±SEM) is shown on the left (*P <0.05, n.s. not significant, two-tailed paired Student's *t* test).

Accumulating evidence suggests that ERV-derived regulatory elements govern the expression of cellular factors (72). We therefore used the Genomic Regions Enrichment of Annotations Tool (GREAT) (47) to predict cellular pathways that may be *cis*-regulated by HIV-1-induced ERVs. We used the CAGE-filtered ERV set for this analysis since ERVs harboring TSS are more likely to regulate the expression of adjacent genes and since their activation is less likely to be the result of read-through transcription. GREAT analyses revealed that ERV elements activated by all three HIV-1 clones tested were primarily found in the vicinity of annotated immunity genes (Figure 4A). For example, genes involved in ‘defense response to virus’, ‘type I IFN signaling pathway’ or ‘negative regulation of viral genome replication’ may be regulated by ERV repeats that are activated in HIV-1 infected CD4^+^ T cells. This includes well-characterized immunity genes such as *ADAR*, *OAS2*, *IFIT1*, *IFI16*, *IFITM1*, *IFITM3* and *TRIM22* that are known to encode for proteins with broad antiviral effects (57). In addition to immunity-related gene sets, infection with the HIV-1 clone STCO1 activated ERVs in the vicinity of genes involved in ‘constitutive secretory pathway’, ‘regulation of endocytic recycling’ and ‘receptor recycling’.

In some cases, fusion transcripts of host genes with HIV-1-induced ERV sequences were observed. For example, the transcripts of *DHRS2* and *SEMA4D* were fused to upstream LTR12D and LTR12C sequences, respectively, and upregulated upon HIV-1 infection (Supplementary Figure S2). DHRS2/HEP27 is an NADPH-dependent dicarbonyl reductase that stabilizes p53 (58) and promotes cellular senescence (59), while SEMA4D/CD100 is an immunoregulator that is associated with T cell exhaustion in HIV-1 infected individuals (60–62). Thus, both factors may be involved in the exhaustion of the immune response and increased senescence upon HIV-1 infection.

Two additional notable examples for potentially *cis*-regulated targets are the guanylate-binding proteins 2 and 5 (GBP2 and GBP5). The respective genes are located directly downstream of LTR12C solo-LTRs (21221_LTR12C and 21276_LTR12C) and part of a larger gene cluster that emerged as a result of several duplication events (63) (Figure 4B). GBP2 and GBP5 are large IFN-inducible GTPases that exert broad antiviral activity as they suppress viral glycoprotein maturation by inhibiting the cellular protease furin (35). In mock infected CD4^+^ T cells, *GBP2* transcription is initiated downstream of the LTR12C element, resulting in the production of an 11-exon transcript (Figure 4C, top). Upon HIV-1 infection, however, an alternative TSS start site within the 3′ half of the LTR12C is activated. Although this novel transcript variant harbors an additional exon, it is predicted to encode the same GBP2 protein since the additional exon lacks putative start codons. In case of *GBP5*, a 12-exon mRNA starting within the LTR12C repeat is produced in both HIV-1 infected and uninfected cells (Figure 4C, bottom). Upon infection, however, transcription of the LTR12C element is upregulated about 2-fold by all three HIV-1 clones tested. Together, these findings strongly suggest that HIV-1-induced LTR12C elements modulate the expression of GBP2, GBP5 and potentially other host factors involved in antiviral immunity.

### The LTR12C repeats upstream of *GBP2* and *GBP5* act as promoters

To further elucidate the role of HIV-1-induced ERVs in regulating cellular immune responses, we focused on GBP2 and GBP5, since these two factors restrict diverse viral pathogens, including HIV-1, measles virus, Zika virus and highly pathogenic IAV (35). Furthermore, the LTR12C element upstream of *GBP2* (21221_LTR12C) was the most strongly induced ERV locus harboring a transcription start site (Figure 3B).

**Figure 5.**
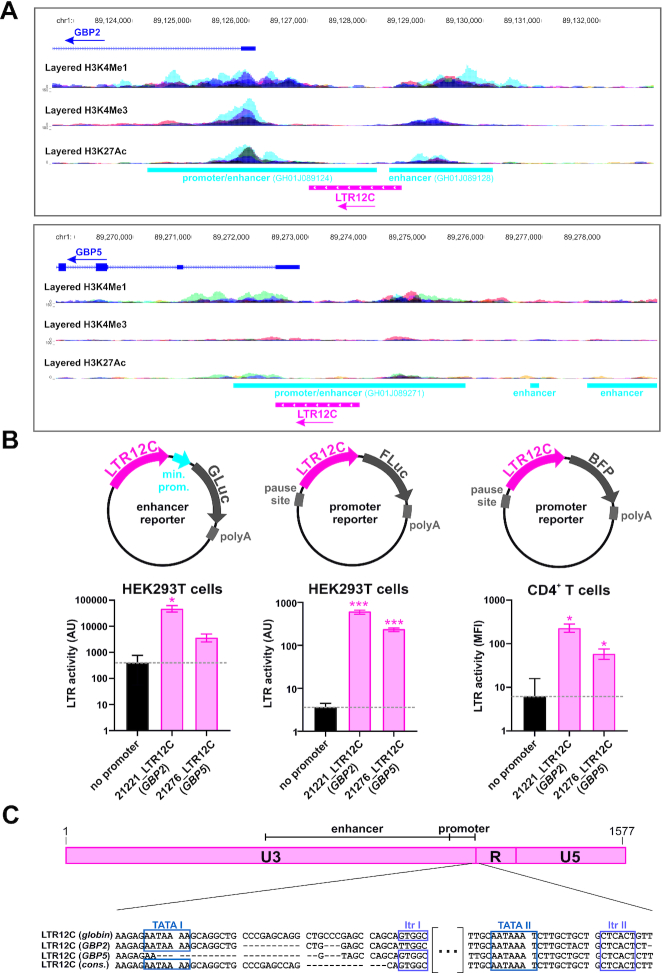
The LTR12C repeats upstream of *GBP2* and *GBP5* act as promoters. (**A**) Promoter and enhancer elements overlapping with the LTR12C repeats upstream of *GBP2* (top) and *GBP5* (bottom). Chromosomal positions are indicated on top, *GBP* genes are shown in dark blue, LTR12C repeats are highlighted in pink and promoter/enhancer sequences identified by GeneHancer (66) are shown in light blue. Histone modifications frequently found near promoters (H3K4Me3) or enhancer elements (H3K4Me1, H3K27Ac) are shown as three individual tracks and were obtained from the Encyclopedia of DNA Elements (ENCODE) via the UCSC browser (98). An overlay of data obtained from seven different cell types (GM12878, H1-hESC, HSMM, HUVEC, K562, NHEK, NHLF) is shown. The tracks show data from the Bernstein lab at the Broad Institute, as part of the ENCODE Consortium. (**B**) LTR12C repeats upstream of *GBP2* or *GBP5* were inserted into enhancer (left) or promoter (middle, right) reporter vectors. Schematic vector maps are shown on top. In the left and middle panels, HEK293T cells were co-transfected with the indicated reporter vectors expressing *Gaussia* luciferase (GLuc) or firefly luciferase (FLuc) and control vector. Two days post transfection, reporter luciferase activity was determined and normalized to the activity of the control luciferase. In the right panel, primary CD4^+^ T cells were electroporated with the indicated reporter vectors expressing blue fluorescent protein (BFP). Two days post electroporation, the mean fluorescence intensity (MFI) of BFP cells was quantified by flow cytometry. In (B), mean values of 3–18 independent experiments ± SEM are shown (*P < 0.05; *** *P* < 0.001). (**C**) The overall structure of the consensus LTR12C (1577 nt) is schematically illustrated on top. At the bottom, partial LTR12C sequences upstream of the globin locus, *GBP2* and *GBP5* were aligned with the consensus sequence. Two pairs of TATA box and initiator (Itr) sequence are indicated by blue and violet frames, respectively. The 5′ TATA box (TATA I) and initiator (Itr I) have previously been shown to initiate transcription in the LTR12C repeat upstream of the globin locus.

Since LTR12C repeats had previously been shown to act as enhancer elements regulating the expression of cellular genes in the globin (64) and olfactory (65) gene locus, we hypothesized that this may also be the case for the LTR12C elements upstream of *GBP2* and *GBP5*. In line with this, both LTR12C repeats overlap with enhancer and promoter sequences identified by GeneHancer (66) and are flanked by histone marks (H3K4Me1, H3K4Me3 and H3K27Ac) typically associated with regulatory elements (Figure 5A) (67). These histone marks were observed in several cell types, including B lymphocytes, embryonic stem cells, myoblasts, endothelial cells, fibroblasts and keratinocytes (Figure 5A) (68), as well as primary CD4+ T cells, the main target cells of HIV-1 (Supplementary Figure S3) (69).

To test the enhancer activity of these two solo-LTRs, we inserted them into a reporter plasmid expressing *Gaussia* luciferase under the control of a minimal promoter. Indeed, insertion of 21221_LTR12C(*GBP2*) and (to a lesser extent) 21276_LTR12C(*GBP5*) increased *Gaussia* luciferase expression (Figure 5B, left panel). Previous CAGE studies and mutational analyses identified a GTGGC initiator motif (Itr) that serves as TSS in many LTR12C repeats (55, 70), although it does not match the consensus Itr sequence BBCABW (where B = C/G/T and W = A/T) (71). This site is located about 30 nucleotides downstream of an AATAAAA motif that has been shown to act as TATA box in an LTR12C element regulating globin expression (72–74). While the 21276_LTR12C(*GBP5*) locus harbors a deletion in this TATA-box and 21221_LTR12C(*GBP2*) contains a mutation in the adjacent initiator sequence, a similar pair of AATAAAT sequence and Itr consensus can be found further downstream in both 21221_LTR12C(*GBP2*) and 21276_LTR12C(*GBP5*) (Figure 5C). This raised the possibility that the solo-LTRs upstream of GBP2 and GBP5 act as promoters and are sufficient to drive gene expression. Indeed, both LTRs induced the expression of firefly luciferase and BFP reporter genes in the absence of any other promoter element (Figure 5B, middle and right panels, Supplementary Figure S4A). This was the case in HEK293T cells, as well as primary CD4^+^ T cells.

Notably, RNA sequence reads obtained from HIV-1 infected primary CD4^+^ T cells strongly suggest that transcription is initiated around the second TATA box (Supplementary Figure S4B) that is flanked by several consensus Itr sequences and intact in both the 21221_LTR12C(*GBP2*) and 21276_LTR12C(*GBP5*) element (Figure 5C, Supplementary Figure S4B). Together, these data demonstrate that the LTR12C/*GBP* repeats harbor functional core promoter elements and are sufficient to initiate transcription of the downstream antiviral genes *GBP2* and *GBP5*.

### LTR12C repeats are associated with strong responsiveness of *GBPs* to cytokine stimulation

**Figure 6.**
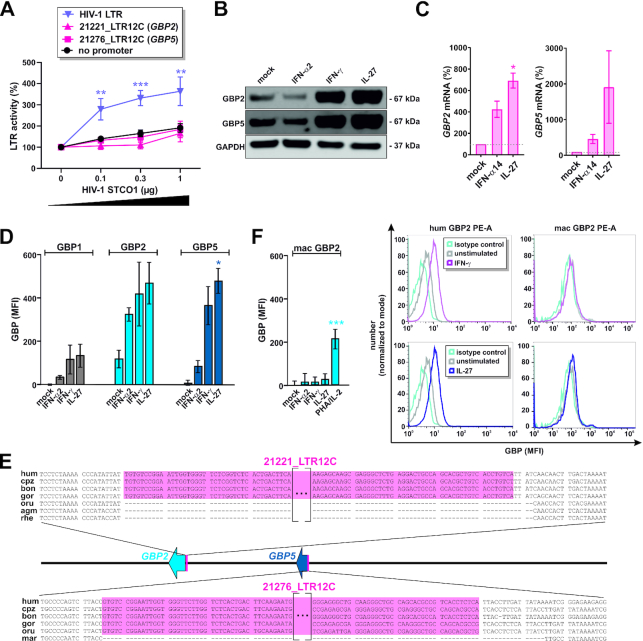
LTR12C repeats are associated with responsiveness of *GBPs* to cytokine stimulation. (**A**) HEK293T cells were co-transfected with the indicated firefly luciferase promoter reporter plasmids, a *Gaussia* luciferase control vector and increasing amounts of the HIV-1 STCO1 clone. Two days post transfection, luciferase activities were determined. Mean values of 4–9 independent experiments ± SEM are shown. (**B**) Primary human CD4^+^ T cells were stimulated with IFN-α2 (500 U/ml), IFN-γ (200 U/ml) or IL-27 (5 ng/ml) for 3 days or left untreated, and GBP2 and GBP5 expression was analyzed by Western blotting. One representative Western blot is shown. (**C**) Primary human CD4^+^ T cells were stimulated with IFN-α14 (50 ng/ml) or IL-27 (5 ng/ml) for 3 days or left untreated, and *GBP2* and *GBP5* mRNA levels were determined by qPCR. Mean values of three independent donors ± SEM are shown. (**D**) Primary human PBMCs were stimulated with IFN-α2 (500 U/ml) IFN-γ (200 U/ml) or IL-27 (50 ng/ml) for 3 days or left untreated, and GBP1, GBP2 and GBP5 expression was analyzed by flow cytometry. Mean values of three independent donors ± SEM are shown. (**E**) Alignment of the LTR12C integration sites upstream of *GBP2* and *GBP5* in humans (hum), chimpanzees (cpz), bonobos (bon), gorillas (gor), orangutans (oru), African green monkeys (agm), rhesus macaques (rhe) and marmosets (mar). (**F**) Primary rhesus macaque PBMCs were stimulated with IFN-α2 (500 U/ml), IFN-γ (200 U/ml) or IL-27 (50 ng/ml) for 3 days or left untreated, and GBP2 expression was analyzed by flow cytometry. Mean values of four independent donors ± SEM are shown. (*P <0.05; ** *P* <0.01; *** *P* <0.001). Exemplary primary FACS data for GBP2 induction upon IFN-γ and IL-27 treatment are shown on the right.

The mechanisms triggering activation of transposable elements in HIV-1 infected cells are still poorly understood. Previous studies reported that the lentiviral proteins Tat and Vpr are sufficient to activate HERV-K and LINE-1 transposable elements, respectively (75–78). To investigate whether any HIV-1 protein may directly activate LTR12C promoters, we co-transfected HEK293T cells with firefly luciferase reporter constructs for HIV-1 LTR or the LTR12C repeats upstream of *GBP2* and *GBP5* and a proviral construct encoding HIV-1 STCO1. In contrast to infected primary CD4^+^ T cells, transfected HEK293T cells lack important sensing pathways and do not mount an IFN response upon transfection of proviral HIV-1 DNA (79). As expected, HIV-1 increased HIV-1 LTR-driven gene expression in a dose-dependent manner (Figure 6A). In contrast, the LTR12C solo-LTRs were not activated above background levels. These findings suggest that HIV-1 stimulates LTR12C promoters indirectly, for example by activating cellular transcription factors.

Two previous studies found that the activation of transcription start sites in LTR12C repeats is associated with the number of GATA and TATC motifs that serve as binding sites for the transcription factor GATA-2 (52,80). 21221_LTR12C(*GBP2*) and 21276_LTR12C(*GBP5*) harbor 5 putative GATA-2 binding sites each. However, over-expression of GATA-2 had no significant effect on LTR12C-driven gene expression (Supplementary Figure S5A), while it activated the promoter of *iASPP*, a known target of GATA-2 (48).

In 2017, Chuong and colleagues reported binding of the immuno-regulatory transcription factors IRF1 and/or STAT1 to the LTR12C repeats upstream of *GBP2* and *GBP5* (10), suggesting that they may be activated by pro-inflammatory cytokine signaling. Since HIV-1 infection increased IFN-γ expression by 62%±14% (p_adj_ = 0.004) in our RNA-seq experiment (40), we hypothesized that this cytokine may be involved in LTR12C-driven GBP expression in HIV-1 infected cells. In line with this, stimulation of primary CD4+ T cells with IFN-γ potently induced the expression of GBP2 and GBP5 (Figure 6B) (34,35). IL-27, which is known to activate an IFN-γ-like STAT1 response (81) also strongly enhanced GBP2 and GBP5 expression, while IFN-α was less effective (Figure 6B, C). Intriguingly, GBP1, which lacks an upstream LTR12C element (Figure 4B) and does not restrict HIV-1 (35), is less responsive to cytokine stimulation (Figure 6D, Supplementary Figure S5B).

To further elucidate the role of LTR12C repeats in the cytokine responsiveness of GBPs, we directly compared peripheral blood mononuclear cells (PBMCs) from humans and rhesus macaques (*Macaca mulatta*). In contrast to humans, macaques lack the entire *GBP5* gene and do not harbor an LTR12C element upstream of *GBP2* (Figure 6E) as this family of solo-LTRs can only be found in apes (Supplementary Figure S5C). Control experiments confirmed that both human and macaque GBP2 can be efficiently detected by flow cytometry (Supplementary Figure S5D). In contrast to its human ortholog, however, macaque GBP2 was not upregulated in response to IFN-α, IFN-γ or IL-27 (Figure 6F), although rhesus cells showed a typical induction of ISGs upon IFN stimulation (Supplementary Figure S5E). In summary, these findings suggest that the presence of LTR12C elements upstream of *GBP2* and *GBP5* determine the cytokine responsiveness of these two antiviral genes.

### IFN-γ responsive elements enhance LTR12C-driven *GBP* expression

**Figure 7.**
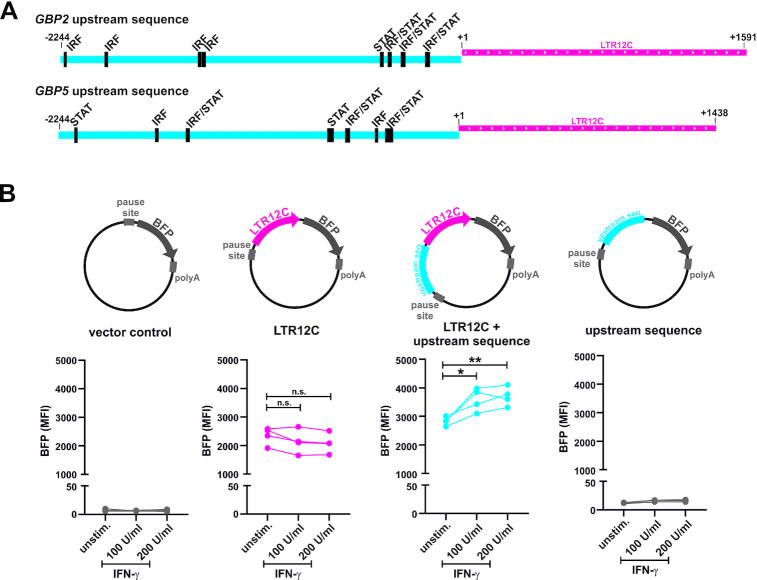
IFN-γ responsive elements enhance LTR12C-driven *GBP* expression. (**A**) IRF and STAT binding sites in the LTR12C elements of *GBP2* (top), *GBP5* (bottom) and upstream enhancer sequences as predicted by *Homo sapiens* Comprehensive Model Collection (HOCOMOCO) (82). (**B**) HEK293T cells were co-transfected with the BFP reporter plasmids indicated at top and a GFP control vector. Cells were stimulated with the indicated amounts of IFN-γ or left untreated, and BFP MFI were determined two days later. The results of three to four independent experiments are shown (*P <0.05; ** *P* <0.01; n.s. not significant).

To further elucidate the role of LTR12C in the inducibility of GBP expression, we searched the LTR12C repeats upstream of *GBP2* and *GBP5* for putative IRF and STAT binding sites, using the HOmo sapiens COmprehensive MOdel Collection (HOCOMOCO). This tool comprises a collection of transcription factor binding models, based on several thousand ChIP-Seq experiments (82). While no IRF and STAT binding sites were identified within the LTR12C elements themselves, clusters of IRF and STAT target motifs were found directly upstream (Figure 7A). These clusters overlap with annotated enhancer regions (Figure 5A), suggesting that they may modulate LTR12C-driven gene expression upon IFN stimulation. To test this hypothesis, we generated BFP reporter vectors that harbor either the LTR12C repeat of *GBP2* alone or in combination with the upstream sequence harboring predicted IRF and STAT bindings sites (Figure 7B, top). While the LTR12C repeat alone did not respond to stimulation, IFN-γ induced the combination of LTR12C and upstream enhancer sequence from humans in a dose-dependent manner (Figure 7B, bottom). Notably, the upstream sequence alone (without any LTR12C promoter) was not sufficient to drive reporter gene expression (Figure 7B, right). Cytokine inducibility was confirmed in primary CD4^+^ T cells, where IFN-γ and IL-27 boosted LTR12C-driven gene expression by 10–60% in the presence of the upstream sequence harboring IRF and STAT bindings sites (Supplementary Figure S6). Together, these findings suggest that regulatory elements upstream of the LTR12C repeat enhance LTR12C-driven GBP2 expression upon cytokine stimulation.

### GBP2 and GBP5 restrict HERV-K (HML-2) Env

While GBP2 and/or GBP5 can be found in many mammalian species (63), LTR12C elements are evolutionarily younger and unique to certain apes (53,83,84) (Supplementary Figure S5C). Thus, the combination of LTR12C promoter and antivirally active GBP may have provided a selection advantage in the evolutionary arms race with ancient viruses infecting early *Hominoidea* species. One potential ancient target of GBP-mediated restriction is HERV-K (HML-2). This retrovirus was still replicating in our direct ancestors when LTR12C elements got fixed in our ancestors’ genomes (53,83–85). Ultimately, HERV-K (HML-2) was also endogenized, and its exogenous form went extinct.

**Figure 8.**
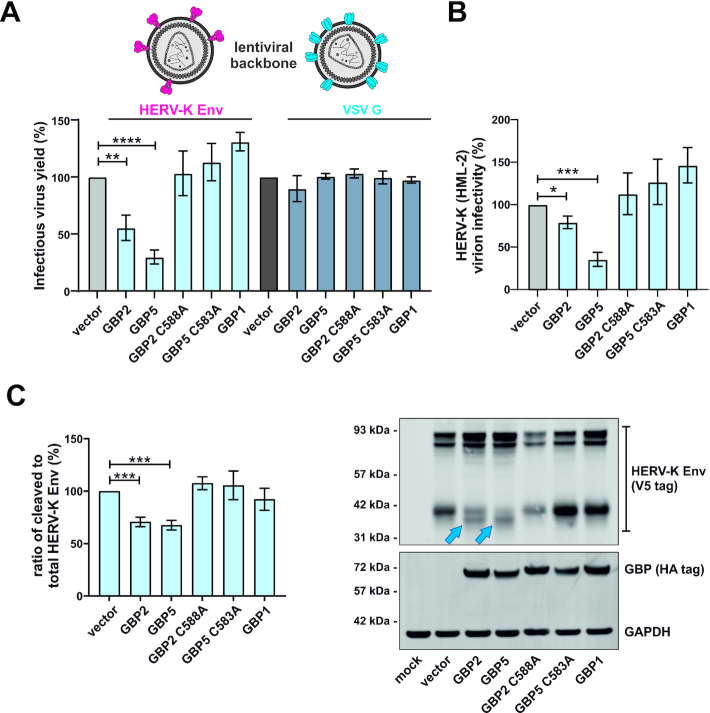
GBP2 and GBP5 interfere with HERV-K (HML-2) maturation and reduce HERV-K (HML-2) pseudovirion infectivity. (**A**) HEK293T cells were co-transfected with an *env*-deficient lentiviral luciferase reporter construct (pSIvec1ΔenvLuc) and expression plasmids for HIV-1 Rev, the indicated GBPs and either HERV-K Env Δ659–699 (left) or VSV G (right). Two days post transfection, cell culture supernatants were harvested, and infectious pseudovirion yield was determined by infecting CRFK cells and quantifying luciferase activity three days later. Mean values of 2–3 independent experiments ± SEM are shown (**P <0.01; **** *P* <0.0001). A cartoon illustrating HERV-K and VSV pseudoparticles is shown on top. (**B**) To calculate HERV-K pseudovirion infectivity, total infectious yield of the samples shown in (A) was normalized to the amount of p27 capsid as determined by ELISA. Mean values of 3 independent experiments ± SEM are shown (**P <0.01; **** *P* <0.0001). (**C**) HEK293T cells were described in (A). Two days post transfection, cells were harvested and expression of HERV-K Env, GBP and GAPDH was determined by Western blotting. Blue arrows indicate a shift in the electrophoretic mobility of HERV-K Env in the presence of GBP2 and GBP5. Cleavage efficiency of HERV-K Env was quantified by determining the ratio of cleaved to total Env. The quantification of 4 independent blots ± SEM is shown on the left (** *P* <0.01; *** *P* <0.0001; **** *P* <0.0001). One representative Western blot is shown on the right.

The LTR promoters of HERV-K (HML-2) harbor several NF-κB and IRF responsive elements suggesting that infection with this extinct retrovirus induced pro-inflammatory cytokine responses (86,87). Furthermore, reconstruction of its Envelope (Env) protein revealed that it is activated by furin (49) and may therefore be sensitive to GBP2/5-mediated restriction. To test this hypothesis, we produced lentiviral luciferase reporter viruses harboring HERV-K (HML-2) Env in HEK293T cells over-expressing GBP2/5 or not. Indeed, GBP5 and (to a lesser extent) GBP2 significantly reduced total infectious virus yield, while two mutants thereof and their paralog GBP1, which do not inhibit furin (35), showed no such antiviral activity (Figure 8A, left). Notably, the inhibitory activity of GBP2 and GBP5 is Env-dependent as virions harboring the glycoprotein of vesicular stomatitis virus (VSV G) instead of HERV-K (HML-2) Env are resistant to GBP-mediated restriction (Figure 8A, right) (35). In line with impaired furin-mediated activation of Env, GBP2 and GBP5 reduced relative virion infectivity (Figure 8B), which was determined by normalizing total infectious virus yield to the amount of capsid protein in the supernatant of the virus-producing cells. Finally, Western blotting confirmed that GBP2 and GBP5 interfere with HERV-K (HML-2) Env maturation as they reduced the ratio of cleaved mature Env to total Env and resulted in a shift of its electrophoretic mobility (Figure 8C). These findings strongly suggest that the IFN-inducible proteins GBP2 and GBP5 have already protected our ancestors from retroviral infections.

## DISCUSSION

Hosts are constantly co-evolving with viruses. As a consequence of the tremendous selection pressure exerted by viral pathogens, host cells have evolved sophisticated means to sense and restrict viral replication. This includes the evolution of transcription factors regulating antiviral gene expression upon infection. However, viruses adapted to this hostile environment and managed to efficiently subvert infected cells for their own purposes. For example, retroviruses such as HIV-1 acquired the ability to hijack cellular transcription factors such as NF-κB that are activated upon infection to drive viral gene expression via their LTR promoters (88). Here, we add another level to this ongoing arms race, demonstrating that expression of the antiviral host factors GBP2 and GBP5 is driven by endogenous retroviral LTR12C promoters that got fixed in the human genome and were coopted by the host.

Combining an unbiased RNA-sequencing approach with mechanistic analyses, we demonstrate that these solo-LTRs harbor transcription start sites that are activated upon HIV-1 infection and govern the expression of GBP2 and GBP5. This regulatory mechanism has also implications beyond HIV-1, since GBP2 and GBP5 suppress the replication of diverse viruses by inhibiting the virus-dependency factor furin (34,35). This includes major human pathogens such as IAV, measles virus, Zika virus (35) and potentially also SARS-CoV-2, which harbors a furin cleavage site in its spike protein (89,90). In addition to that, we demonstrate that GBP2 and GBP5 are not only active against currently circulating viral pathogens, but may have already protected our ancestors as they also suppress the maturation of the envelope protein of the extinct retrovirus HERV-K (HML-2).

Although the human genome comprises hundreds of thousands of endogenous retroviral repeats, we found that infection of primary CD4^+^ T cells with HIV-1 primarily increases the transcription of ERV9 family members, including LTR12C solo-LTRs. This finding is in agreement with two previous publications that also observed increased transcription of ERV9 repeats upon HIV-1 infection (14,52). While these studies did not investigate the functional consequences of ERV9 induction, we uncovered a role of these HIV-1 responsive ERVs in regulating antiviral gene expression. We show that several HIV-1-induced ERVs may be involved in regulating IFN responses and other immunity pathways. Consistent with this, ERV9 solo-LTRs (i.e. LTR12, LTR12_, LTR12B-F) are enriched in *cis*-regulatory elements (91), provide numerous TFBS (54) and are known for the presence of conserved TSS (13,52,73,80).

While most LTR12C-initiated transcripts are non-coding (80), some of them are fused to down-stream cellular genes (9). Indeed, we identified ERV-gene fusion transcripts that are initiated within LTR12C elements and strongly induced upon HIV-1 infection. Among others, our RNA-seq results revealed that HIV-1 infection induces LTR12C-initiated transcripts encoding the antiviral factors GBP2, GBP5 (34,35) and the immunoregulator SEMA4D (60–62). Further characterization of the LTR12C repeats upstream of *GBP2* and *GBP5* demonstrated that they do not only harbor TSS, but act as *bona fide* promoters and are sufficient to drive downstream gene expression. Previous CAGE studies and mutational analyses identified a TATA box and adjacent initiator site that are conserved in the 3′ halves of many LTR12C repeats (55,70). Notably, however, this TATA box is deleted in the LTR12 promoter of *GBP5*, and our RNA sequencing reads suggest that transcription of *GBP2* and *GBP5* is initiated ∼90 bp further downstream. In line with the occurrence of several alternative transcription initiation mechanisms, the LTR12C repeat regulating ZNF80 expression harbors multiple TSS (92). This variation between individual LTR12C elements may at least in part be a result of their high CpG frequency (93) and the resulting high mutation rate (94).

Since *GBP2* and *GBP5* represent prototypic IFN-γ stimulated genes, we hypothesized that they may not only act as promoters, but also mediate responsiveness to cytokine stimulation. To our surprise, the LTR12C elements alone did not respond to IFN-γ stimulation. However, IFN-γ stimulation induced reporter gene expression as soon as upstream enhancer sequences harboring canonical STAT and IRF binding sites were present. These findings indicate that basal LTR12C-driven GBP expression can be further enhanced by IFN-γ responsive elements directly upstream of the LTR12C repeat. Future studies mapping the exact determinants of IFN responsiveness are highly warranted to further elucidate the interplay of LTR12C promoters and enhancer elements in the regulation of GBP expression. Notably, HIV-1 infection induced the expression of IFN-γ in our RNA-seq study (40), suggesting that HIV-1 may at least in part activate LTR12C-driven GBP2 and GBP5 expression via an increased release of IFN-γ. This mechanism would also result in a protective induction of LTR12C-driven GBP2/5 expression in uninfected bystander cells. Furthermore, other pathogens triggering an IFN-γ response may result in a similar activation of the LTR12C repeats upstream of *GBP2/5*. Nevertheless, additional studies are required to elucidate why HIV-1 infection primarily results in the activation of ERV9 repeats and whether other LTR12C loci are also regulated by IRF and/or STAT binding enhancer elements.

We would also like to point out that HIV-1 infection resulted in a more pronounced activation of the LTR12C repeat upstream of *GBP2* (∼8-fold increase) in comparison to the LTR12C element upstream of *GBP5* (∼2-fold increase), and only the former reached statistical significance. Mutational analyses of these two LTR12C elements and their upstream sequences may therefore help to identify determinants of HIV-1 responsiveness. Furthermore, it will be important to define the relative contribution of LTR12C-driven gene expression to total GBP2 and GBP5 levels in uninfected and infected cells, e.g. by characterizing LTR12C knockout cells. Although knockout of specific LTR12C elements is challenging due to their repetitive nature, this experimental approach would also allow to investigate whether the induction of LTR12C-driven GBP2/5 expression upon HIV-1 infection has a significant effect on viral replication.

The key role of GBP2 and GBP5 in the defense against invading pathogens is further highlighted by our finding that they prevent the activation of the envelope glycoprotein of HERV-K (HML-2). This ancient retrovirus integrated into the germ line multiple times between 35 and one million years ago (95), when also ERV9 entered the genome (53,83,84) and primate lentiviruses were already circulating (96,97). Notably, the LTR12 elements upstream of *GBP2* and *GBP5* are most likely the result of two independent integration events since the common *GBP* ancestor duplicated before the fixation of LTR12C repeats in primates (53,63,83,84). Furthermore, the LTR12C promoter of *GBP2* is absent from orangutans and therefore probably younger than that of *GBP5*, which emerged before the divergence of orangutans and *homininae*.

So why was an LTR12C element fixed independently upstream of two closely related antiviral factors? More than 5000 LTR12 solo-LTRs are present in the human genome (44). Most likely, many more integration events occurred but never got fixed in the population as they had neutral or detrimental effects on host fitness. Notably, immunity genes may be particularly prone to retroviral integration events as they are usually activated upon viral infection. Thus, it is tempting to speculate that the LTR12C repeats regulating *GBP2* and *GBP5* expression are few of many solo-LTRs that ultimately got fixed in the human population as they provided a selection advantage to their host. In line with this, ERVs and other transposable elements have recently emerged as the primary source of novelty in gene regulatory networks in primates (91).

In summary, our results uncovered an example of how our immune system exploits relics of once infectious retroviruses to regulate immune responses in HIV-1 infected cells. Furthermore, our findings highlight the emerging role of retroviral enhancer and promoter elements in modulating cellular gene expression. Future studies will reveal whether infections with other viral pathogens also trigger LTR-mediated immune responses and provide important insights into the role of endogenous retroviruses in the spread and pathogenesis of viral infections.

## DATA AVAILABILITY

The RNA-Seq data have been uploaded to the Gene Expression Omnibus (GEO) database (accession number #GSE117655). Representative primary flow cytometry data and Western blots are included in the manuscript and the supplementary figures. Supplementary Tables list log2-fold changes in the expression of ERV families (Supplementary Table S2), unfiltered individual ERV loci that are upregulated at least 0.5 log2-fold upon HIV-1 infection (Supplementary Table S3) and CAGE-filtered ERV loci (Supplementary Table S4).

## Supplementary Material

gkaa832_Supplemental_Files
