# Supplementary material for: HIV-1 infection activates endogenous retroviral promoters regulating antiviral gene expression
Source: Nucleic Acids Res. 2020 Oct 6;48(19):10890–908. doi: 10.1093/nar/gkaa832 (PMC7641743; doi:10.1093/nar/gkaa832)
Supplement: gkaa832_Supplemental_Files [file gkaa832_supplemental_files.zip › Supplementary Figures S1-S6 and Table S1.pdf]

HIV-1 infection activates endogenous retroviral promoters regulating antiviral gene expression

Smitha Srinivasachar Badarinarayan[1], Irina Shcherbakova[2], Simon Langer[1,3], Lennart Koepke[1], Andrea Bloetz[1], Dominik Hotter[1], Frank Kirchhoff[1], Konstantin M. J. Sparrer[1], Gunnar Schotta[2], Daniel Sauter[1,*]

[1]Institute of Molecular Virology, Ulm University Medical Center, Ulm, 89081, Germany
[2]Ludwig Maximilians University and Munich Center for Integrated Protein Science (CiPSM), Biomedical Center, Planegg-Martinsried, 81377, Germany
[3]Sanford Burnham Prebys Medical Discovery Institute, La Jolla, CA, 92037, USA

SUPPLEMENTARY FIGURES S1-S6 and Table S1

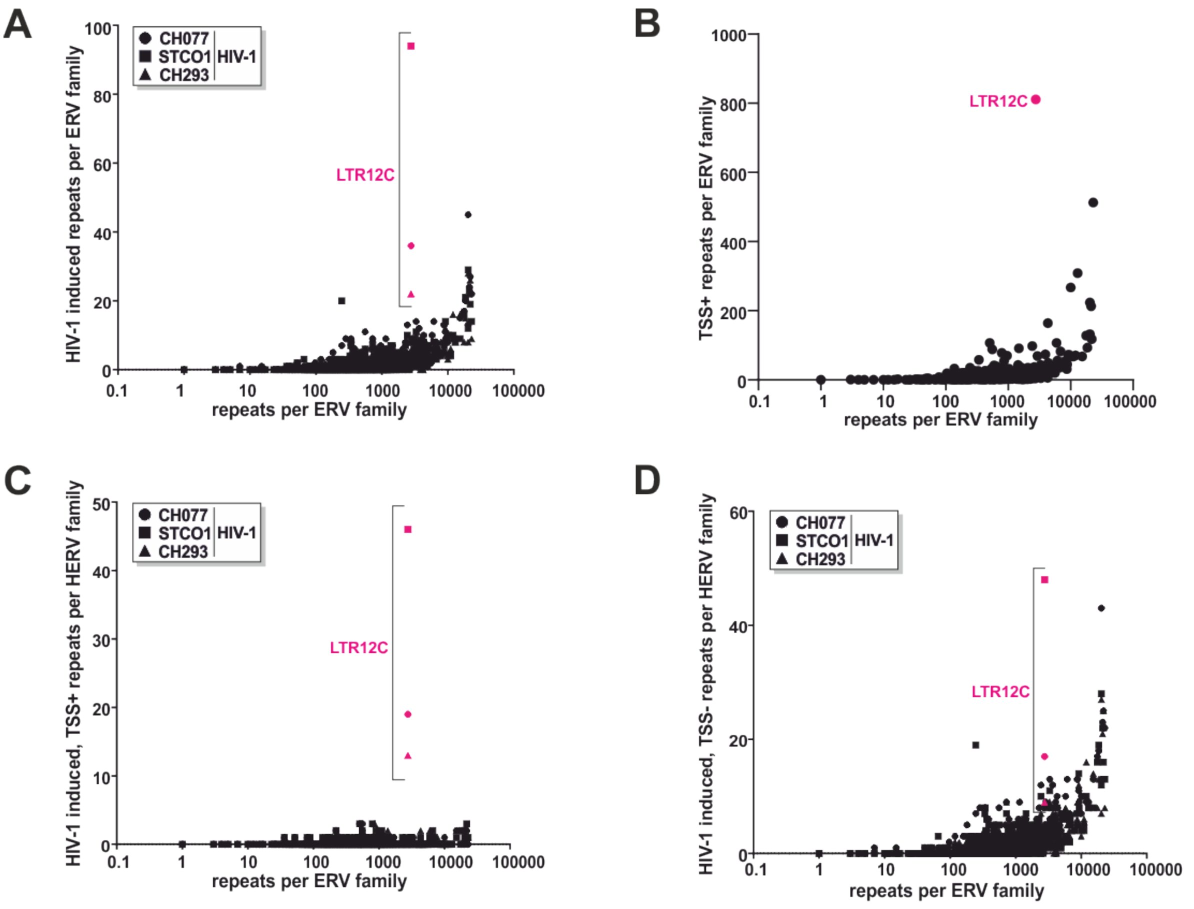

E

RepeatID	chromosome	start	end	width	strand
723311_MLT1J	chr22	39490029	39490480	452	-
21276_LTR12C	chr1	89272452	89273890	1439	-
578311_LTR12C	chr13	110820975	110822522	1548	+
428069_LTR21C	chrX	64850687	64851086	400	+
592267_MER4E1	chr14	71512603	71513198	596	+
163736_LTR12C	chr4	6672540	6673747	1208	+
404512_MER54B	chr9	114902837	114903617	781	+
20225_LTR9	chr1	84583111	84583698	588	+
390852_LTR2	chr9	62800850	62801305	456	+
348497_MamGyp-int	chr8	26507740	26508856	1117	-
646937_MLT1A1	chr17	40552782	40553192	411	+
533029_ERV3-16A3_I-int	chr12	62252040	62253373	1334	-
328295_LTR12C	chr7	104939974	104941430	1457	+
428070_LTR21B	chrX	64850818	64851088	271	+
21221_LTR12C	chr1	89127018	89128609	1592	-
722464_MLT1A0	chr22	35660544	35660852	309	-
15333_THE1A	chr1	67207421	67207774	354	+
250311_LTR12C	chr5	126853695	126855085	1391	-
339510_HERVK14C-int	chr7	150561618	150565017	3400	-

RepeatID	chromosome	start	end	width	strand
142880_LTR16C	chr3	119588188	119588637	450	-
202612_LTR12C	chr4	141332214	141333759	1546	-
592723_LTR12_	chr14	73567334	73568063	730	+
607537_HUERS-P1-int	chr15	44877921	44882678	4758	-
42779_Harlequin-int	chr1	206053000	206053902	903	+
478914_HERV9N-int	chr10	89406838	89412211	5374	+
489992_MLT1C	chr11	5709775	5710190	416	-
373833_MER92-int	chr8	125952376	125953678	1303	+
600849_MLT1D	chr14	103136805	103137010	206	-
623392_LTR12C	chr16	16103819	16105191	1373	-
587307_MER101-int	chr14	50756556	50757011	456	+
521591_LOR1-int	chr12	9951519	9951756	238	+
701614_LTR10B1	chr20	49281110	49281576	467	-
241951_LTR12C	chr5	95850982	95852649	1668	+
31439_LTR12C	chr1	154670567	154671998	1432	+
722463_MLT1B	chr22	35660111	35660521	411	+
718748_MLT1B	chr22	17777408	17777764	357	+
142881_LTR78B	chr3	119589242	119589378	137	-

Supplementary Figure S1. LTR12C repeats are over-represented among ERV elements induced upon HIV-1 infection. Number of individual repeats in each ERV family vs number of **(A)** HIV-1 induced repeats, **(B)** TSS harboring repeats, **(C)** HIV-1 induced repeats harboring TSS and **(D)** HIV-1 induced repeats harboring no TSS. **(E)** List of the 37 CAGE-filtered ERV loci that were upregulated by all three HIV-1 clones tested (see also Figure 3D). LTR12C elements are highlighted in pink.

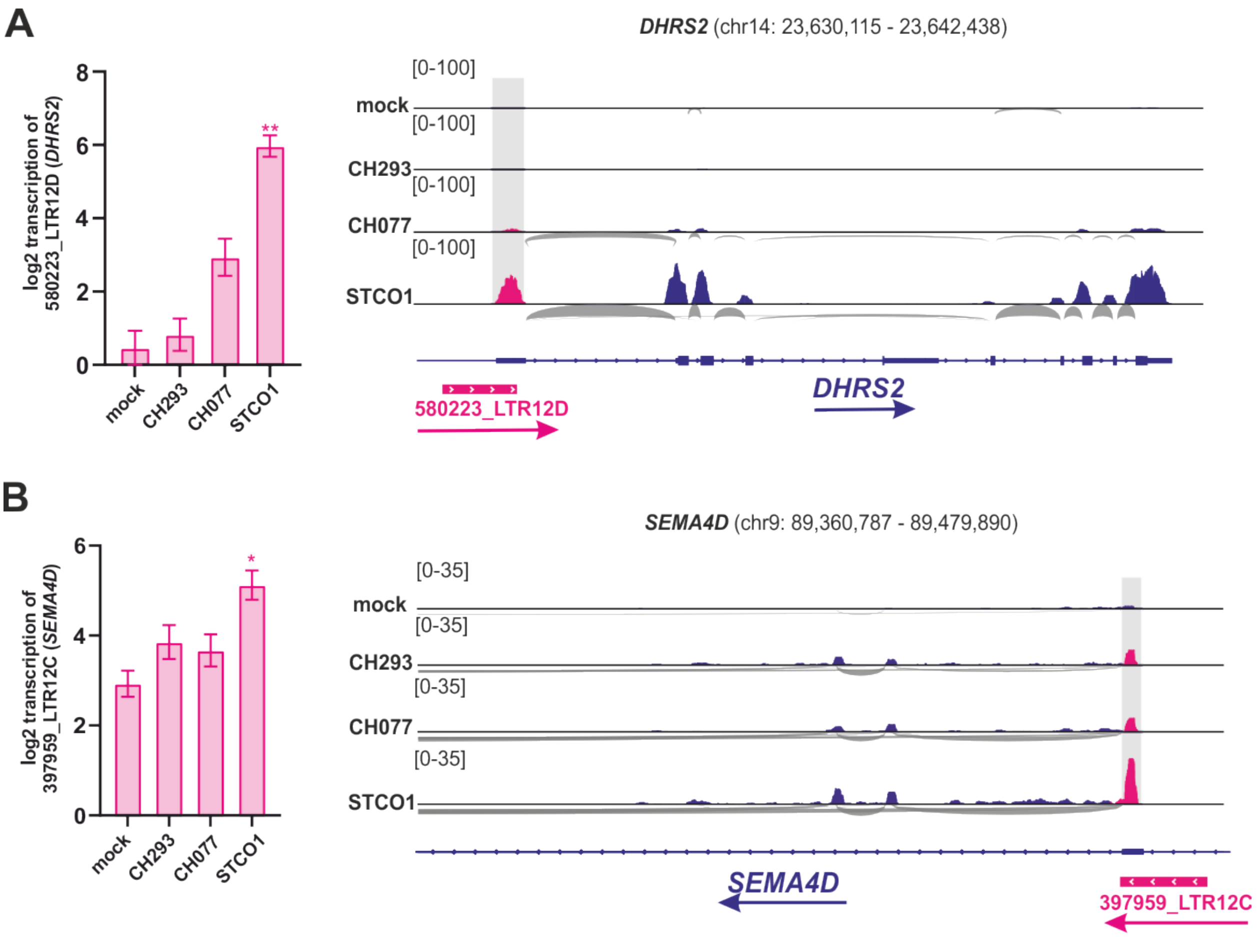

Supplementary Figure S2. HIV-1 infection triggers the expression of LTR12D/C elements upstream of *DHRS2* and *SEMA4D*. (A, B) Representative RNA-Seq data for the expression of (A) *DHRS2* and (B) *SEMA4D* including the respective upstream solo-LTRs are shown for mock and HIV-1 STCO1 infected CD4^{+} T cells. LTR12C and LTR12D are highlighted in pink. Sequence coverage and exon linkage are shown in blue and grey, respectively. The mean transcription of LTR12C/D in all four donors for all three viruses tested (±SEM) is shown on the left (* $p < 0.05$; ** $p < 0.01$).

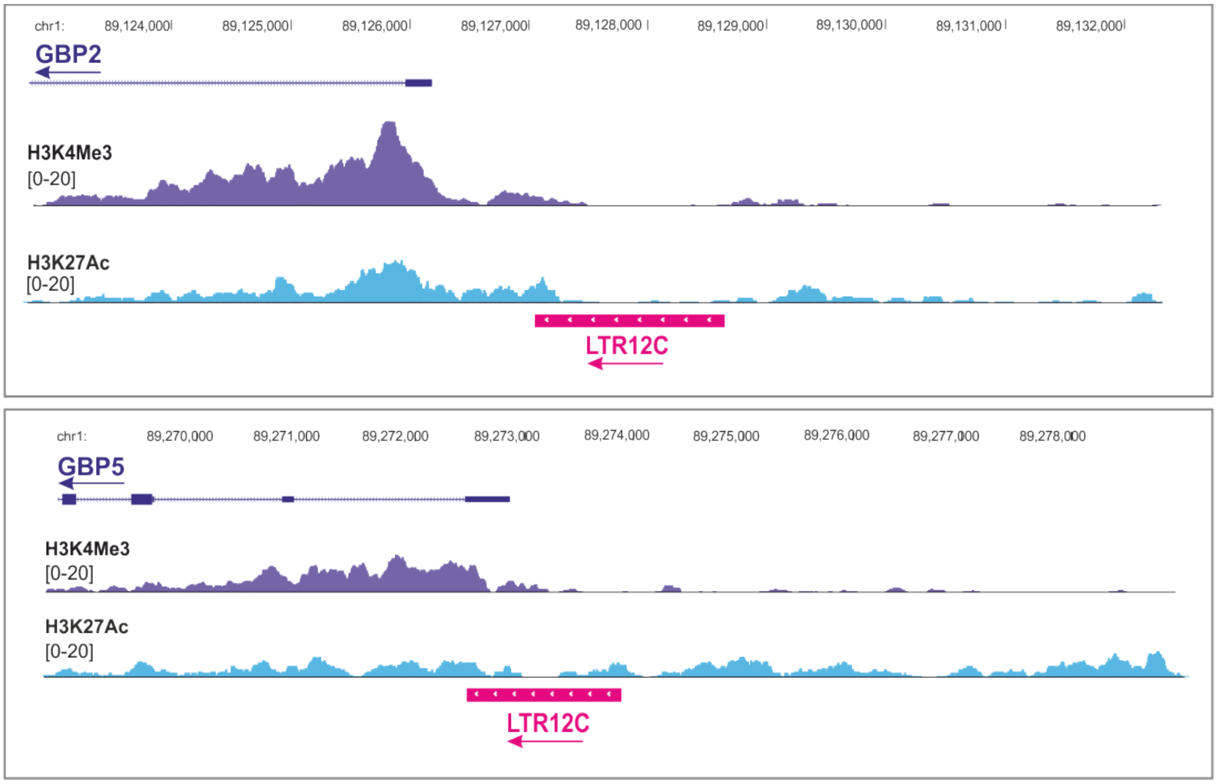

Figure S3. Histone modifications of 21221_LTR12C (*GBP2*) and 21276_LTR12C (*GBP5*) in primary CD4^{+} T cells. Chromosomal positions are indicated on top, *GBP* genes are shown in dark blue, LTR12C repeats are highlighted in pink. H3K4Me3 and H3K27Ac modifications in primary CD4+ T cells are shown as two individual tracks. Data are derived from GEO: GSM3486210 (H3K4me3_bio1: SRR8235450; H3K27ac_bio1_1: SRR8235444).

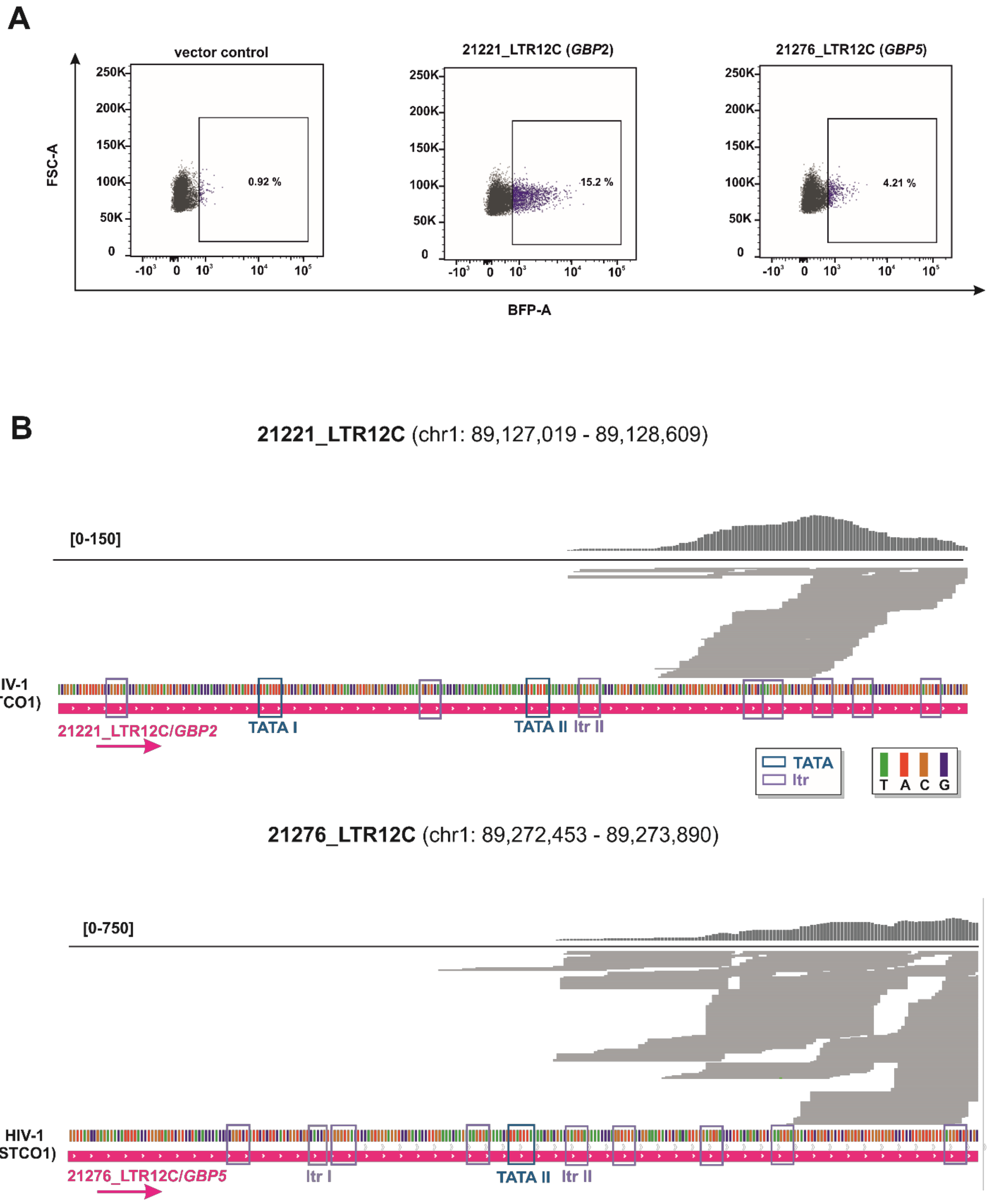

Supplementary Figure S4. Initiation of *GBP2* and *GBP5* transcription in HIV-1 infected $CD4^+$ T cells. (A) Representative flow cytometry data of the $CD4^+$ T cell experiment shown in Figure 5B. **(B)** RNA-Seq reads and coverage in the LTR12C repeats upstream of *GBP2* (top) and *GBP5* (bottom). Representative data of cells infected with HIV-1 STCO1 are shown. TATA boxes and initiator (Itr) sequences are indicated by blue and violet frames, respectively. Sequence coverage and reads are shown in grey. See Figure 5C for exact sequences.

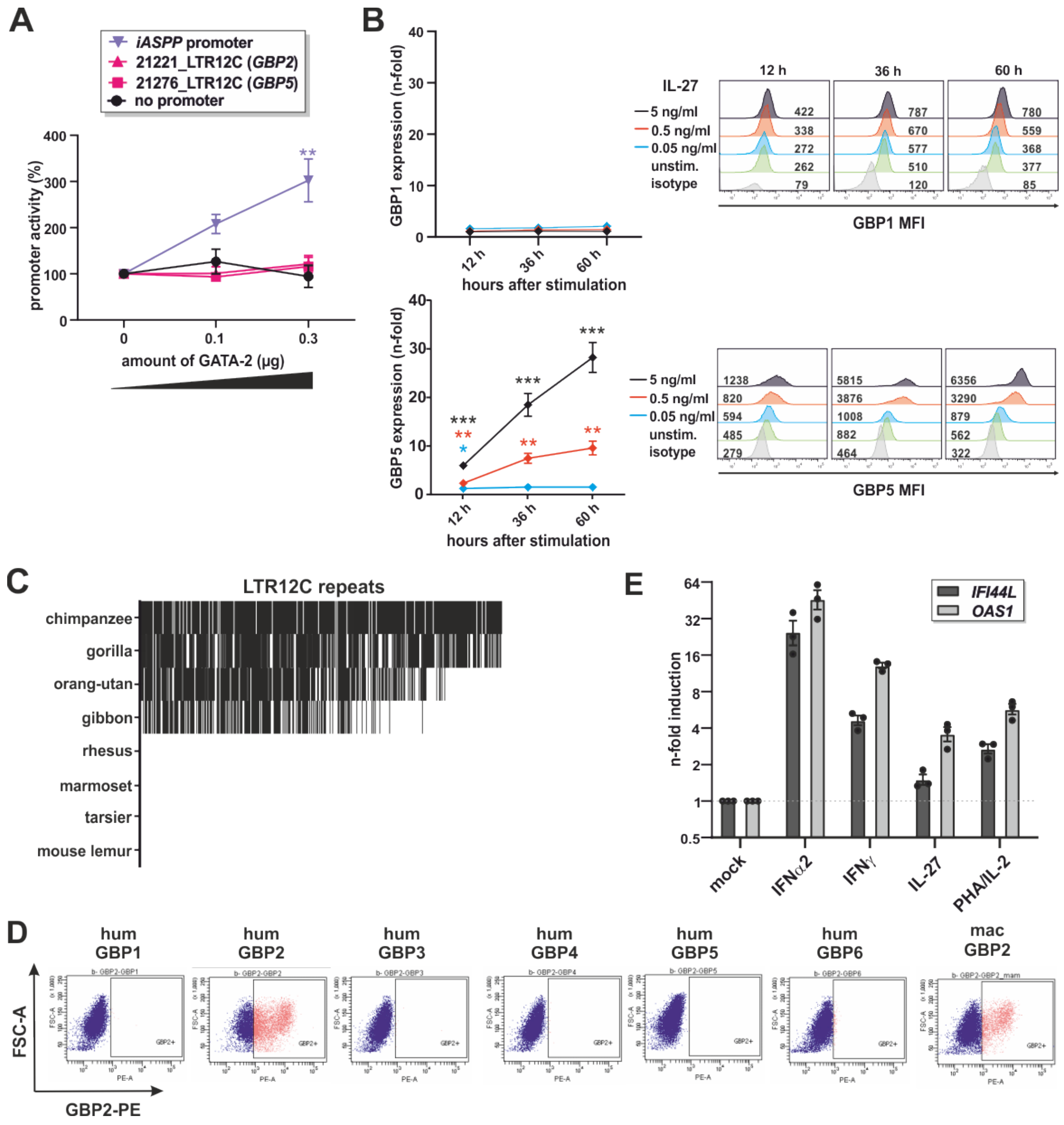

Supplementary Figure S5. LTR12C repeats are associated with responsiveness of *GBPs* to cytokine stimulation (A) HEK293T cells were co-transfected with the indicated firefly luciferase promoter reporter plasmids and increasing amounts of a GATA-2 expression plasmid. Two days post transfection, luciferase activities were determined. Mean values of 3 independent experiments ± SEM are shown. **(B)** Primary human CD4⁺ T cells were stimulated with increasing amounts of IL-27 for the indicated periods of time before GBP1 (top) or GBP5 (bottom) protein levels were quantified by flow cytometry. Mean values of 6 independent donors ± SEM are shown on the left. Representative primary data are shown on the right. **(C)** Presence of human orthologous LTR12C copies in the reference genomes of the indicated primate species as obtained via http://herv-tfbs.com (91). **(D)** HEK293T cells were transfected with plasmids co-expressing BFP and the indicated GBPs. Two days post transfection, the specificity of an anti-GBP2 antibody was analyzed by flow cytometry of BFP positive cells. Representative primary data are shown. (* p <0.05; ** p <0.01; *** p <0.001). **(E)** Primary rhesus macaque PBMCs were stimulated with IFN-α2 (500 U/ml), IFN-γ (200 U/ml), IL-27 (50 ng/ml), or PHA/IL-2 (1 µg/ml and 10 ng/ml) for 3 days or left untreated. *IFI44L* and *OAS1* mRNA levels were determined via qPCR. Representative results obtained with one donor measured in technical triplicates ± SEM are shown.

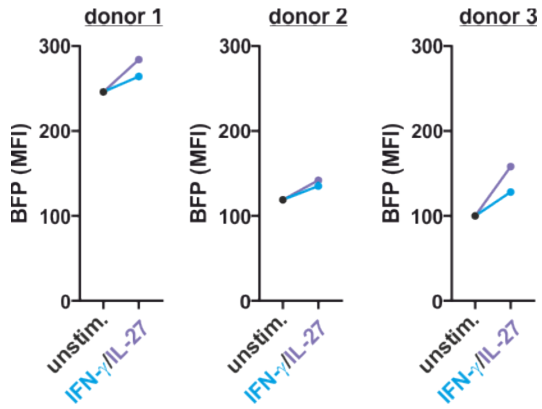

Supplementary Figure S6. Effects of IFN-γ and IL-27 on 21221_LTR12C(*GBP2*)-driven gene expression in primary CD4+ T cells

PHA/IL-2 treated CD4+ T cells were electroporated with a reporter construct containing 21221_LTR12C(*GBP2*) in combination with its upstream sequence that harbors predicted STAT and IRF binding sites. 1 day later, cells were stimulated with IFN-γ (200 U/ml), IL-27 (50 ng/ml) or left untreated. 1 day later, BFP expression was determined by flow cytometry. Background reporter gene expression was determined using an empty vector control and subtracted. Results of three independent donors are shown.

Supplementary Table S1. Primers used to generate firefly luciferase/BFP reporter constructs

Construct name	Primer name	Primer sequence
No promoter F.luc	fw	CGGAGCTCACCATGGAAGATGCCAAAAACATTAAG
	rev	CGGAATTCTTACTTGTACAGCTCGTCCATGCCGGG
No promoter BFP	fw	CGGAGCTCACCATGGTGAGCAAGGGCGAGGAGC
	rev	CGGAATTCTTACTTGTACAGCTCGTCCATGCCGGG
pGL4.32 LTR12C GBP2 F.luc	fw	CGGCTAGCTGACAGGTGACAGCGTGCTGGCAG
	Rev- overlap with Fluc	GGCATCTTCCATGGTGTGTCCGGAATTGGTGGGTTCTCGG
	fw – overlap with LTR12C_GBP2	CAATTCCGGACACACCATGG AAGATGCCAAAAACATTAAG
	rev	CGGAATTCTACACGGCGATCTTGCCGCCCTTC
pGL4.32 LTR12C GBP2 BFP	fw	CGGCTAGCTGACAGGTGACAGCGTGCTGGCAG
	Rev- overlap with BFP	CCTTGCTCACCATGGTGTGTCCGGAATTGGTGGGTTCTCGG
	fw – overlap with LTR12C_GBP2	CAATTCCGGACACACCATGGTGAGCAAGGGCGAGGAGCTG
	rev	CGGAATTCTTACTTGTACAGCTCGTCCATGCCGGG
pGL4.32 LTR12C GBP5 F.luc	fw	CGGCTAGCTGAGAGGTGACGGCGTGCTGGCAGC
	Rev- overlap with Fluc	GTTTTTGGCATCTTCCATGGTGTCCGGAATTGGTG
	fw - overlap with LTR12C_GBP5	CAATTCCGGACACCATGGAAGATGCCAAAAACATTAAG
	rev	CGGAATTCTACACGGCGATCTTGCCGCCCTTC
pGL4.32 LTR12C GBP5 BFP	fw	CGGCTAGCTGAGAGGTGACGGCGTGCTGGCAGC
	Rev - overlap with BFP	GCCCTTGCTCACCATGGTGTCCGGAATTGGTGGGTTCTTG
	fw - overlap with LTR12C_GBP5	GAACCCACCAATTCCGGACACCATGGTGAGCAAGGGCGAGG AGC
	rev	CGGAATTCTTACTTGTACAGCTCGTCCATGCCGGG
pGluc mini TK2+ hum LTR12C GBP2	fw	CGCTCGAGTGACAGGTGACAGCGTGC
	rev	CGCAAGCTTCATGGTGTGTCCGG
pGluc mini TK2+ hum LTR12C GBP5	fw	CGCTCGAGTGAGAGGTGACGGCGTGC
	rev	CGCAAGCTTGTGTCCGGAATTGG
pGL4.32 hum upstream only GBP2 with LTR12C	fw	CGGCTAGCTGGGTTGTCAGAC
	rev – overlap with BFP	CCTTGCTCACCATGGTGTGTCCGGAATTGGTGGGTTCTCGG
	Fw- overlap with hum upstream element	CAATTCCGGACACACCATGGTGAGCAAGGGCGAGGAGCTG
	rev	CGGAATTCTTACTTGTACAGCTCGTCCATGCCGGG
pGL4.32 hum upstream only GBP2 without LTR12C	fw	CGGCTAGCTGGGTTGTCAGAC
	rev – overlap with BFP	GCTCCTCGCCCTTGCTCACCATGGTTATCAACAACTTGACTA AAATACCTC
	Fw- overlap with hum upstream element	GTCAAGTTGTTGATAACCATGGTGAGCAAGGGCGAGGAGCT G
	rev	CGGAATTC TTA CTTGTACAGCTCGTCCATGCCGGG
